# Outlines of Comparative Anatomy

**Published:** 1835-10-01

**Authors:** 


OUTLINES OF COMPARATIVE ANATOMY. By *Robert E. Grant*, M.D., &c. Professor of Comparative Anatomy in the University of London. Parts 1 and 2, 8vo. Bailliere, London.

THE appearance of this work constitutes an era in the history of anatomy and physiology in this country, and will, we doubt not, give an impetus to the pursuit of this interesting and important study, which has been too much neglected in this country—one reason, probably, why physiology, as taught in many of our schools, remains disfigured by many crude theories and speculations, which have long since disappeared on the Continent; tested, as they have been there, by the rigid laws deduced from the successful pursuit of comparative anatomy, embryogeny or organogenesy, and teratology, or the science of monstrosities. The co-ordinate pursuit of these departments of science has given birth to the doctrine of morphology, or the unity of organic elements, which, under the various forms which they assume in different classes and genera of animals, is the very foundation of the French transcendental, or philosophic school of anatomy. The doctrines of this school have hitherto, unfortunately, made but little progress in this country, and, indeed, have but too often met with the ridicule of those who have occupied our professorial chairs, a circumstance which can only be attributed to the generally-prevailing ignorance of the facts upon which the science rests. The work of Dr. Grant, however, will, we doubt not, do much to dissipate this ignorance, deeply imbued, as its author evidently is, with the doctrines of the philosophic school, and familiar as he proves himself to be with the facts upon which those doctrines are founded. From this circumstance, the "Outlines" of our author stand agreeably contrasted with the elementary works on this science, which the mere English reader has hitherto had in his hands. The works of Monro, Fyfe, and even Blumenbach, improved as the system of the latter was by Lawrence and Coulson, are scarcely more than the records of a number of facts, apparently capable of no generalization, and shewn to follow no general and uniform laws.

It is to the constant reference to such general laws that the present work owes its superiority, which cannot fail to render the study of comparative anatomy not only more attractive, and therefore more general, but also more useful; calling into exercise, as it will do, the reasoning powers of the mind, where hitherto too often the memory only was exercised.

Doubtless, many of our readers possess the course of lectures on this subject, which appeared last year in the Lancet, and hence may think it unnecessary to possess themselves of the "Outlines;" to beginners however in the science, we would say, study the "Outlines" first, and, having obtained the elements of the science from this work, you will be able to avail yourselves with much greater benefit of the more extensive view of the subject presented to your view in the admirable and elaborate course of lectures to which we have alluded.

In the first part of the work, our author, commencing with the organs of relation, treats of those of support, of attachment, and of motion; or, in other words, of the osseous, the ligamentous, and the muscular systems; the latter of these subjects, however, is not proceeded far with in this part. The plan which our author adopts, is that of commencing with the very lowest forms of animal organization, and exhibiting to his readers the traces which are there met with of the systems under review; he then successively takes up the different classes in the ascending scale, and shews not only the gradual additions made to the several organs, in passing from the less perfect to the more perfect and finished forms, but also the various modifications which the organs undergo, in order to suit them to the peculiar habits and functions of different animals. Finally, the organs pass under review as they exist in the most perfect of all animal forms—in man himself, in whom indeed each organ is not seen to attain *specifically* its most perfect development—a circumstance which would be in the highest degree incongruous, and present to our view a monstrous form far stranger than any of those which Nature occasionally exhibits to us; but in whom that harmonious balancement of the organs, that perfect equipoise, is met with, in which we perceive the most perfect *general* development of all the organs; that is to say, each organ, or rather perhaps each class of organs, assumes the most perfect form that is compatible with a corresponding degree of development in the other organs.

We will proceed to give our readers some idea of the manner in which our author handles his subject, and at the same time to introduce a curious and interesting subject to their notice. The fifth section of the first chapter treats of the organs of support in the vertebrated classes, in which our author introduces the skeleton to the view of his readers in the following words:—

"The most constant and first-formed part of the skeleton is the vertebral column, which is composed of moveable vertebræ, each of which consists of several elements that are found most isolated and distinct in the lowest classes, and in the embryo state of the highest. The elements which appear most constant and distinct in the composition of a vertebra are the round central body, or *cyclo-vertebral* element, the two superior laminæ or *peri-vertebral* elements which encompass the spinal chords, the two portions of the superior spinous process, or the *epi-vertebral* elements, the two inferior laminæ, or *para-vertebral*

elements, which form a cavity for the blood-vessels, and the two portions of the inferior spinous process, or the *cata-vertebral* elements. The cyclo-vertebral elements are tubular in the articulated classes of animals where they envelope the whole trunk as hollow segments, they are nearly solid to their centre, and present two concave surfaces in fishes; they are convexo-concave in reptiles, and have flat surfaces in mammalia. They are the most constant and typical parts of the vertebral column. The other vertebral elements vary their forms and positions chiefly according to the dimensions of the organs they embrace, and the extent of surface required for muscular attachment; consequently they vary much in different parts of the same column, and in the skeletons of different classes. In the caudal portion of the skeleton of an osseous fish they are designed to give great extension for the attachment of the powerful lateral muscles which move the tail. The body of the vertebra, or cyclo-vertebral element, supports the two superior laminæ or peri-vertebral elements, which early unite above to form the small foramen for the spinal cord; and beyond their termination we observe the interspinous bone and the ray of the external fin, which are the two epi-vertebral elements placed in a vertical line. The analogous elements are seen on the lower part of the vertebra, where the two inferior laminæ or para-vertebral elements form a large foramen for the lodgment of the great continuation of the aorta above, and the vena cava below. The inferior interspinous bone, and the ray of the external fin, are the two cata-vertebral elements placed in a vertical line, like the epi-vertebrals above. These vertebral elements often assume, in the region of the abdomen in fishes, another position; thus the superior elements remain as before indicated, but the inferior laminæ or para-vertebrals are stretched out in a horizontal direction, and have the two cata-vertebrals extended from their ends in form of a pair of ribs to encompass the organs of this part of the trunk. The vertebral elements situate above the body of the bone expand in the region of the head in the same manner as we here see those below the cyclo-vertebral element in the region of the abdomen; and this they do in order to encompass the soft parts contained in the cavity of the skull and in the face. The epi and peri-vertebrals are most expanded in the skull and the sacrum, and the para and cata-vertebrals, where they embrace the viscera of the trunk." 59.

The above views with respect to the vertebra are adopted by our author from M. Geoffroy de St. Hilaire, who has published an interesting paper on the subject in the Mémoires du Muséum.* They form a favourite doctrine of the transcendental school, and notwithstanding the ridicule which has been attempted to be cast upon it in this country, it may be regarded as one of the best established of those doctrines. Although it was G. H. Hilaire who gave the definite and philosophical view to the theory, with which we find it regarded in the pages of our author, yet the doctrine of the skull consisting of a series of vertebral pieces highly expanded and developed, was first somewhat incidentally glanced at by J. P. Frank, in the year 1792, in his Delectus Opusculorum Medicorum.

"I have always been of opinion," says he, "that every vertebra of the spinal column is to be regarded as a small transverse cranium. The last, and most conspicuous and moveable of all the vertebræ is that called the calvaria or skull-cap.†"

This view was taken up and further developed by Oken, and has since

---

* Mémoires du Muséum, vol. 9.

† Delect. Opuscul. Medic. Vol. II. p. 8.

been advocated with various modifications, by Dumeril, Spix, Blainville, Carus, Meckel, Cuvier, and Burdach, with many others. Amongst the first of the supporters of this doctrine, we find the Shakespeare of Germany—Goëthe,* who seems to have had a great predilection for the study of analogies, and who may be regarded as the very father of vegetable morphology, by which every part of the flower and the fruit is shewn to consist merely of a modification of the leaf.

These various authors, however, whilst agreeing in the general doctrine of the skull consisting of a series of vertebral pieces, are not unanimous as to the number of such pieces entering into its composition. Oken, Dumeril, Spix, Carus, Cuvier, and Meckel, all unite in assigning three as the number of the cranial vertebræ, whilst Bojanus regards them as four; Hilaire, however, who, as we have seen, has investigated the subject more closely, perhaps, than any other anatomist, concludes that the skull is composed of seven vertebral pieces. Hence we find our author, who appears closely to follow the last distinguished comparative anatomist in his views on this subject, observes that—

"The number of distinct osseous pieces in the composition of the skull is greatest in fishes, and they correspond nearly with the theory of this part of the skeleton, being composed of seven vertebræ, each consisting, as usual, of a body, with four elements above, and four elements below." 63.

For ourselves, however, we must confess that we have always been inclined rather to follow the first of these views, which certainly accords better with what we see in the human cranium, in which we can readily trace the existence of a series of three vertebral pieces, which are thus indicated by Cuvier:—"Le crâne se subdivise comme en trois ceintures, formées—l'anterieure par les deux frontaux et l'ethmoide; l'intermédiaire par les parietaux et le sphénoide; la posterieure par l'occipital."†

In this view, we cannot see any thing inconsistent with a fair and legitimate use of analogical reasoning, supported, as it is, by the facts observed on tracing the modifications of the vertebra and of the cranium through the various classes of vertebrated animals. Some authors, however, have doubtless prevented the general acceptation of the doctrine, by the crude speculations and fanciful hypotheses with which they have disfigured it.

Of this class of authors we should regard, Spix, who, in a large and expensive folio, has treated of this subject. He believes that, in the cranium, is to be found a perfect repetition of the rest of the skeleton, that of the three vertebral pieces entering into its composition; the superior is the "proper cephalic" vertebral piece—the second is the "thoracico-cephalic," and the third the "pelvi-cephalic" piece. In the bones of the face or upper jaw, attached to the middle of these pieces, he traces a repetition of all the bones of the upper extremity; and in the temporal bone and lower jaw, appended to the lower piece, that of all those of the inferior extremity; the alveolar processes of the jaws being said to represent the phalanges of the toes and the fingers, which are tipped with teeth in lieu of nails!‡

---

* Zur Naturwissenschaft, t. i. p. 250.

† Regne Animal, tom. i. p. 63.

‡ Cephalogenesis. Munich, 1815.

As, then, the internal skeleton of the vertebrated classes consists essentially of a series of double rings of bone, giving protection to the central parts of the nervous and the vascular systems, and the great viscera of the trunk, and having the great bulk of the muscles arranged in layers externally, so, also, does the external skeleton of the articulated classes consist of a series of zones or segments, giving a corresponding degree of protection to the same important organs. In these classes, however, the tegumentary system and the skeleton are consolidated together, and the muscles are themselves placed within the vertebral pieces, which here present only one common cavity, from the absence, as we think, of the cyclo-vertebral elements, rather than, as stated by our author, from the "tubular" condition of these elements. The dorsal and the ventral surfaces respectively of the segments of the skeleton, in these classes, appear to us to consist of elements corresponding to the peri- and para-vertebral elements in vertebrated animals, by which names they might appropriately be designated. Indeed, from a somewhat extended consideration of the subject, we are led to the conclusion, that the analogy between the articulated and vertebrated classes is much greater than has yet been generally allowed by comparative anatomists and zoologists; and of which, before we conclude this article, we hope to have convinced such of our readers as have familiarized themselves with the study of comparative anatomy.

If our limits had allowed, we could with pleasure have taken this opportunity of directing the attention of our readers to other interesting and important subjects, connected with comparative osteology, but we have already exceeded the limits which we had appointed for the notice of the first part.

In the second part of the work, our author resumes the subject of the muscular system, which was only commenced in the first part, and traces it through the several gradations it exhibits, in passing through the higher molluscous classes, from the invertebrate to the vertebrate classes—in the latter of which he treats of it successively in fishes, amphibials, reptiles, birds, mammalia, and man himself. As a specimen of the manner in which our author treats this part of his subject, we make the following extract.

"The muscles of *birds* are more red and vascular, more irritable and dense, than in the cold-blooded vertebrata beneath them, and they possess these properties in the greatest degree in the rapacious tribes, where the respiration is greatest, and where all the functions are most energetic. This condition of the muscular system is required in birds, from the lightness of the medium through which they move, and from their quick and long-contiuued movements through that element. The muscles are more feeble, pale, soft, and palatable in the heavy, slow-moving, phytophagous tribes, where the condition of the bones, and most of the other systems, mark an inferior or reptile state of development. The fleshy parts of the muscles are generally short and thick, especially in the arms and legs of birds, and their tendons are generally long, slender, dense, and often ossified, like many cartilaginous parts of the skeleton. The active, heavy, fleshy parts of the muscles being situate, for the most part, on or near the solid trunk of the bird, the extreme parts, so important for progression, are lightened by receiving and supporting only the long, narrow tendons; hence the long, slender legs and the lightness of the arms in this class. As birds have nearly all the same general form and movements, there is remarkable uniformity in the muscular system throughout this class." 165.

In the fourth chapter, Dr. Grant considers the nervous system; and, as the comparative anatomy of this system has assumed, of late, a position of considerable interest, from the definite nature of the additions made to our knowledge respecting it, and from the interesting reference which the subject has to human physiology, we will endeavour to present our readers with as succinct an account as may be of the subject.

That the character of the nervous system is one of primary importance, in determining the rank which animals hold in the scale of development, appears to be generally admitted by comparative anatomists; certain characters distinguish this system, in all the grand divisions of the animal kingdom. The two first grand divisions, of vertebrate and invertebrate animals, have each their peculiar modification of nervous system—thus:

"The great central portion of the nervous system is perforated by the alimentary canal, in the invertebrated classes, either in its middle, as in the radiata and the mollusca, or at its anterior extremity, as in the articulata; but, in the vertebrata, the spino-cerebral axis lies wholly above the digestive cavity, by which it is no where pierced." 180.

The three grand classes or divisions of invertebrate animals have each a characteristic form of the great nervous centre; and, from this circumstance, Dr. Grant has appropriated to each a name expressive of this difference, in lieu of those in general use; thus, the terms cyclo-neurose, diplo-neurose, cyclo-gangliated, and spini-cerebrated, are respectively applied to the radiated, the articulated, the molluscous, and the vertebrated classes of animals. We do not perceive the necessity for this innovation, nor are the terms themselves beyond the reach of fair criticism; but we pass on.

In the lowest animal forms, those which are embraced by the polygastric and poripherous classes of radiated animals—the infusoria and sponges of the older zoologists, no distinct nervous system has yet been met with; but, as our author supposes, this is most likely owing to the transparency of the nervous filaments in these minute animals (the polygastrica), which, from the activity of their functions, may be well concluded to possess the rudiments, at least, of that form of this system possessed by the higher invertebrated classes. "In the poriphera (our author thinks that), the component particles of the nervous and muscular systems are probably diffused through every part of the soft cellular tissue of the body, which possesses the same living properties in every part, and is almost indefinitely divisible without destroying its vitality." In the polypiphera—the zoophyta of the older zoologists, and comprising the corals and madrepores, it is, in the actinia alone, a large and naked polypus, that a nervous system appears hitherto to have been discovered. In this animal, "nervous filaments surround the muscular foot, beneath the stomach, and present minute ganglia in their course, from which nerves pass out to the circumference, and to the muscular folds, which here possess great power of contraction. The same system probably exists in many other closely-allied forms of polypi."

In the two remaining classes of radiate animals—the acalepha, or sea-nettles, and the echinoderma, of which the asterias, or star-fish, may be mentioned as a familiar instance, the nervous system is pretty constantly met with in the form of a circular white cord, surrounding the mouth, with ganglia developed upon it at regular intervals, the number of which ganglia corresponds with that of the radiating segments of the body. Thus the circular, double nervous cord of the beroe pileus, an acalephous animal, is furnished with eight small white ganglia, interposed between the eight longitudinal bands of cilia, which ganglia supply with minute nervous filaments the various parts of the body ; and in the asterias, which, as before stated, belongs to the echinoderma, a similar circular cord around the mouth has five minute ganglia, corresponding to the five rays of most species of this animal. In the highest forms of radiated animals, as the holothuria and the siponculus, the nervous system is beginning to approach that form which constitutes its type in the articulated classes; thus, in the latter of these genera, two minute longitudinal nervous filaments extend backwards from the œsophageal ring, and are developed only on one side of the body, like the abdominal nerves of the helminthoid articulata.

We now come to the consideration of the nervous system in the articulated classes, where, from the striking analogy which recent researches have shewn it to have with that of vertebrate animals, and, hence, with that of man himself, the subject is invested with considerable attraction. The molluscous classes, indeed, have had a higher rank assigned them in the scale of animal organization than the articulated, in consequence of the superior development of their organs of nutrition—of their digestive, circulatory, and respiratory systems. But surely, in a *zoological* classification, the functions of relation, or, in the expressive language of Bichat, "the functions of animal life," ought to have a higher value assigned to them than those of nutrition, which vegetable forms enjoy in common with animals. In the articulated classes, all the organs of relation, the osseous system (if we may so speak of their skeleton), the muscular and the nervous systems, and most of the organs of the senses, have all of them become infinitely more closely assimilated to the more perfect forms which they assume in the vertebrated classes, than is the case in any of the mollusca. We know that, by reversing their order, we should be in no better predicament; the highest class of the mollusca, the cephalopods, would then be separated widely from the fishes, to which they are doubtless very closely united; but we merely draw attention to this circumstance, in order to point out the close analogy that the organs of relation, in the articulata, have with those of the vertebrata. In fine, whilst the molluscous classes, with their superior organs of nutrition, evidently have, through their highest class—the cephalopods, a close affinity with fishes amongst vertebrate animals, so, likewise, the articulated classes, with their more perfect organs of relation, seem to us to stand in full as close, or even closer, relation to reptiles among the same classes, the transitions of which may, we think, be detected, from the helminthoid classes to the ophidian reptiles, and from the entomoid classes, more especially the crustacea, into the class of chelonian reptiles.

These observations having been premised, we pass on to the consideration of the nervous system, in these classes of animals.

The nervous system is here found to consist of a double lengthened cord, extending along the abdominal surface of these animals, under the alimentary canal, and forming a double ganglionic enlargement, for the most part, in correspondence with each segment of their articulated bodies. The ganglionic enlargement of the cord, corresponding to the anterior segment or head of the animal, is seated above the œsophagus, the two lateral portions of the cord, separating as they approach the under surface of this tube, and, after encircling it, unite in forming a ganglion, generally the largest in the body. These ganglia give off as many pairs of nerves to the muscles and other organs of their bodies, the supra-œsophageal serving as the origin of several pair distributed to the eyes, the antennæ, and other organs of sense and motion seated at the cephalic extremity of the bodies of these animals. The development of these ganglia is in the direct ratio of that of the organs to which they are distributed. This is well exemplified in the four classes constituting the helminthoid section of the articulata; thus, in the entozoa and the annelida, which, as they possess either very imperfectly-developed lateral appendices for progressive motion, or else none at all, and, as they are equally deficient in, or imperfectly supplied with, organs of sense at their anterior extremities, so, also, are their supra-œsophageal and abdominal ganglia in an equally rudimentary condition, and, indeed, frequently not perceptible. In the rotifera, however, "where there is a complex muscular apparatus at the anterior extremity of the trunk, for the motion of the numerous large cilia, and another muscular apparatus for the movements of the strong lateral maxillæ, the nervous system is most developed in that part of the body." In these animals, whilst the supra-œsophageal, with its accessory ganglia, are generally distinct and large, the abdominal ganglia are frequently absent. In the cirrhopods, as "in the anatifa, we perceive a slender white nervous ring, surrounding the œsophagus, and sending out small filaments to the surrounding parts, but scarcely forming a perceptible supra-œsophageal ganglion, from the imperfect development of the sensitive and masticating apparatus in these fixed and inverted testaceous, or entomostraceous animals. As the long-jointed and ciliated feet, with their thick muscular haunches, and supporting the bronchiæ at their base, are developed from the sides of the posterior part of the trunk, the ganglia, like the nervous columns which connect them, are large in that part of the body, and correspond in position with the origin of the several pairs of legs."

"The nervous system of the entomoid classes presents only a more developed condition of the same plan of structure presented by this system in the worms, and that of the most elevated insect or crab begins its development with the simplest helminthoid form." 190.

From the greater development of the nervous system in this section of the articulated classes, comprising the myriapods, the insects, spiders, and crustaceous animals, it is here that comparative anatomists have most fully succeeded in unravelling its structure, and have thus advanced comparative neurology to its present degree of completeness.

In the myriapods, as, *e. g.* in the centipede, we do not find much to detain us, their nervous system presenting only a more developed form of that met with in the helminthoid classes. With the metamorphoses which are undergone by insects, their nervous system does not remain unchanged. In their larva state, they generally present thirteen pairs of ganglia, corresponding with the number of the original segments of the body, which supply with nerves the soft tissues of which they are now composed. These ganglia, during the process of development undergone by insects in passing from the larva, through the pupa, to the imago or perfect state, are observed to approximate, as well by the shortening of the cords, as by their assuming a zig-zag arrangement during the pupa state. These changes are more particularly observed in the thoracic portion of the cords and ganglia, which latter afterwards unite together in the situation most suited to send nerves to the yet undeveloped thoracic members. The nature of these changes produced during development, will be understood by our readers on perusing our author's description of the adult condition of the nervous system in the papilio brassicæ, as observed and described twenty years ago by Herold.

" In the imago or perfect condition of the insect, the loose inter-ganglionic portions of the columns, which were zig-gag in the pupa, have assumed a straight and shorter form—the two last pairs (the 12th and 13th) have coalesced into one ganglion, and advanced from their original position—the cineritious matter has disappeared from two pairs of the abdominal ganglia (7th and 8th) without affecting the original origins of their nerves—four pair of ganglia (the 6th and 5th, and the 4th and 3d) have coalesced at two points of the thorax, to supply nerves to the muscles of the legs and wings—the second and first pairs of ganglia have approached in the head, and diminished the diameter of the œsophageal ring, (which was much greater during its voracious larva state)—the cephalic ganglia have enlarged and extended transversely to form the expanded optic lobes." 196.

Remarkable as are the changes which take place in the form of the nervous system in this, and in other lepidopterous and hymenopterous insects, they are not nearly so extensive as those which obtain in the coleoptera, and which have been observed by Straus Durckheim in the melolontha vulgaris.

" In this coleopterous insect, where the adult form of the whole body is very remote from that of a caterpillar or of an annelide, all the ganglia have disappeared from the short round abdomen, and have accumulated in three contiguous masses in the middle of the thorax, from which the nerves radiate to the organs of motion, and extend backwards into all the segments of the abdomen. The cephalic ganglia have also encreased above and below the œsophagus, and the cerebral lobes passing laterally into the large compound eyes, have advanced still more in their development." 196.

We are indeed much surprised to find that our author never once alludes to the admirable researches of Mr. Newport, " on the nervous system of the sphinx ligustri, and on the changes which it undergoes during the metamorphoses of the insect," which have been published in the Philosophical Transactions for 1832 and 1834. These researches are not only highly valuable from their fully bearing out the statements of Lyonet, of Herold, and of Straus Durckheim, but also from the extraordinary precision of their details, are themselves of intrinsic importance. The sphinx ligustri was selected by Mr. Newport for his observations, on account of the more gradual nature of the metamorphoses which it undergoes, thus allowing of more minute and accurate observation, than is afforded by the papilio brassicæ, employed by Herold, in which these changes take place much more speedily. The results in this way obtained by this gentleman appear to us to be much more precise and important than those of the continental labourers in this field, most of whose works we have consulted in express relation to this point. These investigations are equally creditable to Mr. Newport, as they are calculated to raise the British school of comparative anatomy in the estimation of our further advanced continental brethren.

Our limits will not permit us to say more of the nervous system of the arachnida, the next class as we ascend in the scale, than that,

"Like most other parts of the arachnida, it presents an intermediate condition of development betwixt that of most insects and that of the higher crustacea." 198.

In the highest class of articulated animals, the crustacea, the nervous system is variously developed, according to the more or less perfect development of the animals themselves. It however, for the most part, affects two general forms—a lengthened one, like that of myriapods, as in the lobster, (astacus,) and a concentrated one, analogous to what exists in coleopterous insects, as in the crab-tribe, (maia,)—in these latter our author says—

"All the symmetrical ganglia of the columns are generally collected into two masses, the one in the head, and the other in the centre of the cephalo-thorax, and the motor and sensitive columns are almost confined to a nervous band around the wide œsophagus. The anterior of these, or the supra-œsophageal ganglion, is comparatively small in the brachyourous decapods, from the smallness of the cephalic appendices which it supplies with nerves. The infra-œsophageal nervous mass is of great size, consisting of the whole chain of ganglia, which was originally extended along the body behind the œsophagus, and is favourably situated between the haunches of the legs under a strong internal osseous arch, in the centre of the trunk. It sends out numerous branches to the surrounding viscera, and to the five pairs of legs which radiate from around that point; and the columns are prolonged backwards, ramifying along the short slender post-abdomen, as a simple nervous chord." 203.

Having presented our readers with a general view of the anatomy of the nervous system in the articulated classes, we will now turn their attention to what is known of its minute structure, and endeavour to shew to what part of the nervous system in vertebrated animals it is analogous. Very opposite opinions on this latter head have been held by anatomists and physiologists:—thus, Bichat, Reil, and Ackermann, with some others, have regarded the ganglionary cord of insects and the other articulata as analogous to the great sympathetic of man, to which view of the question, by-the-bye, our author does not advert. This doctrine, however, seems to be sufficiently refuted by the distribution of the nerves rising immediately from this double cord, which is almost exclusively to organs of sense and motion, as well as by the existence of what has appeared to many as the rudiments of a distinct system of organic nerves in most of these classes. There is connected with the supra-œsophageal and lateral ganglia, (which latter, from their connexion, seem to have a close analogy to the first cervical ganglia in man,) a nervous filament, having two or three minute ganglia on its trunk, which runs backwards above the œsophagus, and between that and the dorsal vessel, to both of which and the stomach it distributes nerves. This nerve is the recurrent of Lyonet, but by Straus Durckheim,* has in the melolontha vulgaris been regarded as the rudiments of a sympathetic system, and in this opinion our author appears fully to unite. Mr. Newport, however, in the papers referred to, regards this nerve as the analogue of the pneumogastric or par vagum. This question may then be considered as being still sub judice.

---

* Considerations Générales sur l'Anatomie Comparée des Animaux Articulés, &c. Paris, 1828.

The other view with respect to the nature of the central part of the nervous system in these classes, consists in regarding it as the counterpart of the cerebro-spinal system of man, and the other vertebrata, a doctrine, which as our author states, has been long held by Lyonet, Straus, Dufour, and Chiaje, amongst those who have investigated this part of the animal creation more particularly, and to whose names we may add those of Scarpa, Blumenbach, Cuvier, Meckel, and Gall. We will endeavour, as concisely as possible, to point out to our readers the characters of this analogy. That in the insects and higher entomoid classes the supra-œsophageal ganglia—largely developed as they are, and supplying nerves to the organs of sense—the eyes and antennæ, as well as to those of motion—the maxillæ, and even, if our memory do not deceive us, containing, according to Straus Durckheim, ventricular cavities within their substance—are closely analogous to the brain of higher animals, must, we think, be readily acknowledged. But does the ganglionary cord, stretched through their bodies, answer as closely to the spinal cord of the vertebrata? Is its situation the same as that of the medulla spinalis? Does it possess two distinct nervous tracts giving origin to nerves having sentient and motiferous properties? And, if so, how does its minute anatomy bear upon the still undecided question respecting the existence of a distinct respiratory system of nerves? To these questions we will endeavour to give satisfactory answers.

In the first place, with respect to its situation, it occupies the abdominal and not the dorsal surface of the body, but then, says our author—"all the other viscera of the trunk present the same inverted position; the heart-forming portion of the sanguiferous system occupies the dorsal surface," so that the great nervous columns may be regarded as being "extended for protection along the ventral or under surface of the body."

With respect to the structure of these cords, Weber* very satisfactorily shewed that their ganglia had a very precise analogue in the intervertebral ganglia of the vertebrata. These ganglia, with their connecting tracts, would hence seem to correspond very closely with the sensitive columns of the spinal cord of man, which however occupy the dorsal surface of this cord, whilst it is now shewn that the ganglia of the nervous cord of articulated animals occupy the ventral surface of their bodies, and have a nervous tract, without any such enlargements, passing over their superior surface. It appears, from our author, that the existence of these two distinct tracts was known to Lyonet and to Treviranus, the former seventy, and the latter twenty years ago; and he adds that he has "long shewn the same structure to pervade the articulated classes." Thus, then, the tracts of which the spinal cord of insects is composed, participate in that inverted position, in relation to the organs of vertebrata, which we have already shewn to affect their viscera in general.

Mr. Newport, in his admirable paper, which we have again to regret our author has not availed himself of, in the composition of the present chapter, has most satisfactorily shewn the existence of these two distinct tracts in all the entomoid classes, and has given drawings of them, to which we would

---

* Anatomia Comparata Nervi Sympathici. Auctore E. H. Weber. Lipsiæ, 1817.

refer such of our readers as are desirous of prosecuting this interesting subject. The discovery of the motor tract in insects, at least, we believe is the almost indisputable due of this gentleman, as the drawing given by Dr. Grant from Lyonet, to shew that that author was acquainted with the motor tract in the cossus, quite fails in doing so, the inter-ganglionic nerves there represented being quite distinct from the motor nerves of Newport, and evidently answering to what he deems to be respiratory nerves, which we shall now notice, in order to complete the sketch which we have attempted to give of what is known of the neurology of articulated animals.

Between the several pairs of ganglia, in what we have now shewn to be the spinal cord of these animals, pass off on each side from the upper surface of the cord of insects, and most of the entomoid classes, a minute pencil of transverse filaments, distinct from the symmetrical, or moto-sensitive nerves, which pass from each ganglionic space. These transverse nerves have long attracted the attention of anatomists. They are shewn by Mr. Newport to lay above the motor tract, and in many instances to possess small ganglionic enlargements at the point of separation of the nerves. In many instances it is difficult to make out a distinct continuous tract by which the several nerves are united, but this Mr. Newport has accomplished in several instances, more particularly in the carabus, in which likewise the subjacent motor columns are very distinct. This system of nerves forms very remarkable connexions with the rest of the nervous system; thus it is connected by filaments with the lateral ganglia of the head, which we have already alluded to as analogous to the first cervical ganglia of man, and generally each pair of these nerves sends backwards a communicating filament to the adjoining pair of symmetrical moto-sensitive nerves. These transverse, superadded, or, as Mr. Newport designates them from what he supposes to be their function, respiratory nerves, are distributed amongst the tracheæ and dorsal muscles, and the largest branch goes to the trachea coming direct from the spiracula.

Two opinions appear to have prevailed respecting the functions of these nerves, the one of which is, that they are ordinary motor nerves, the other that they constitute the system of the great sympathetic. Mr. Newport does not agree in either of these opinions, but regards them as a respiratory system of nerves.

Dr. Grant appears to be one of those who consider these nerves as motor; at least we come to that conclusion from his employing the two terms respiratory and motor indiscriminately. That, however, they are not ordinary motor nerves is pretty evident, as Mr. Newport well observes, from their possessing very distinct ganglia in several species; whilst he argues that, from their distribution, they must be respiratory nerves.

Their situation and anatomical connexions would certainly rather lead to the idea of their being the analogue of the great sympathetic, but then they are very sparingly distributed to viscera. We would observe, however, that the existence of the respiratory system of nerves in man, as supposed by Sir C. Bell, is far from proved, that the doctrine is altogether repudiated by the best German physiologists, and that the most recent researches and observations tend to refute it. Then the situation of these transverse nerves does not agree with that of the so-called "respiratory tract" of Sir C. Bell, which holds an intermediate position between the motor and sensitive columns.

In order to make our account of the nervous system of invertebrated animals complete, we extract the following passages from the fourth section of the fourth chapter, on the nervous system of the cyclo-gangliated, or molluscous classes.

"The nervous system is distinctly developed and provided with several ganglionic centres, in all the molluscous classes, from the lowest compound forms of *tunicata* to the highest of the *cephalopods*, and notwithstanding the remarkable diversity of form which the animals of this division present, we can trace a certain similarity of character and unity of place in the development of this system, and in its typical forms, throughout all the cyclo-gangliated classes. In the tunicated and conchiferous animals the columns are chiefly disposed beneath the alimentary canal; in the gasteropods and the pteropods they are more equally distributed around the entrance to the stomach; and in the more elevated forms of cephalopods they at length mount to that supra-œsophageal position which they preserve in all the vertebrata where they cease to embrace the alimentary canal." 204.

"The nearest approach to the vertebrated form of the nervous system is that presented by the *cephalopods*, the highest of the mollusca and of all the intervertebrata. The œsophagus still perforates the brain, as in all the inferior classes, but the greatest portion of that organ, and the symmetrical columns prolonged from it, are here placed above the alimentaiy canal. The brain is enclosed in a distinct organized cranial cavity, numerous symmetrical ganglia are developed on the great nervous axis both before and behind that organ, and sympathetic ganglia are observed in the abdominal cavity." 206.

We will now take leave for the present of our author, and in so doing, strongly recommend his work to all such of our readers as are desirous of acquainting themselves with this most interesting and important science, to which medicine is under so many and weighty obligations. If we except Carus' Comparative Anatomy, translated by Mr. Gore, which, however, is too diffuse for beginners, it is the only book in our language, with which we are acquainted, proper to be put into the hands of any one desirous of studying comparative anatomy scientifically.

